# Recanalization of a chronic long segment ICA near-occlusion using a newly designed carotid artery stent

**DOI:** 10.1007/s00234-025-03772-1

**Published:** 2025-09-20

**Authors:** Johannes Hensler, J. Cordt, F. Wodarg, O. Jansen

**Affiliations:** Department of Radiology and Neuroradiology, University Hospital Schleswig-Holstein Campus Kiel, Kiel, Germany

**Keywords:** Carotid stent, Chronic occlusion, Ischemic stroke, Recanalization

## Abstract

A 67-year-old female presented with subacute transient visual disturbances in her left eye and subacute watershed infarctions in the left MCA territory due to a long segment chronic internal carotid artery (ICA) near-occlusion following ICA stenting (CAS). Angioplasty was performed using two newly designed stents (CARESTO^®^ heal, Acandis, Pforzheim, Germany) from the ICA origin into the cavernous segment without complications, with a favorable clinical outcome.

## Background

**Fig. 1:**
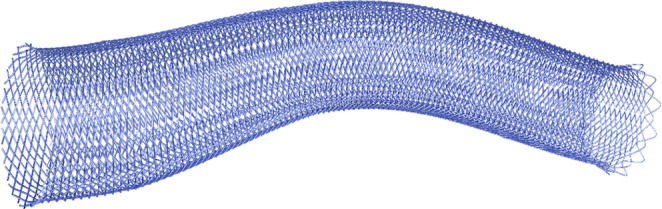
Illustration of a tapered CARESTO Heal stent (schematic) with dense single layer mesh and distal flared ends. The braided Nitinol stent is self-expanding with a complete radiopaque platinum core and a fibrin/heparin coating. © Acandis

Chronic ICA occlusion with or without previous stent-angioplasty presents a therapeutic challenge when patients present with clinical symptoms due to chronic hypoperfusion. In this case, the newly designed CARESTO heal stent **(**Fig. 1**)** was employed to rebuild the artery from its origin to the curve into the cavernous segment. The CARESTO heal is a single-layer, closed-cell stent designed to treat vulnerable plaques with a coverage of up to 40%. Braided nitinol wires with a platinum core provide full-length visibility and maintain the natural course of the vessel. The HEAL coating consists of a thin layer of fully cured fibrin network with covalently bound heparin. As the coating mimics the final step in the natural healing process, it promotes the attachment and colonization of endothelial cells while having anti-thrombogenic and anti-inflammatory effects. Nevertheless, angioplasty of a chronic near-occlusion requires careful interdisciplinary indication and good post-procedural patient management.

## Case presentation

A 67-year-old female presented to our emergency department with recurrent left episodes of amaurosis fugax (1–2 times per day) lasting for six weeks and one episode of acute aphasia. Bilateral CAS (CASPER, Microvention, CA) had been performed nine months earlier due to high-grade restenosis following endarterectomy on the right and high-grade asymptomatic stenosis of the left ICA. After three months of dual antiplatelet therapy, the patient was on apixaban therapy due to a diagnosis of atrial fibrillation. Apart from arterial hypertension, she had no risk factors for arteriosclerosis.

## Investigations

Clinical examination revealed a positive right pronator drift test. Computed tomography angiography (CTA) suggested a long-segment chronic occlusion of the left ICA, accompanied by subsequent subacute and chronic watershed infarctions.

**Fig. 2:**
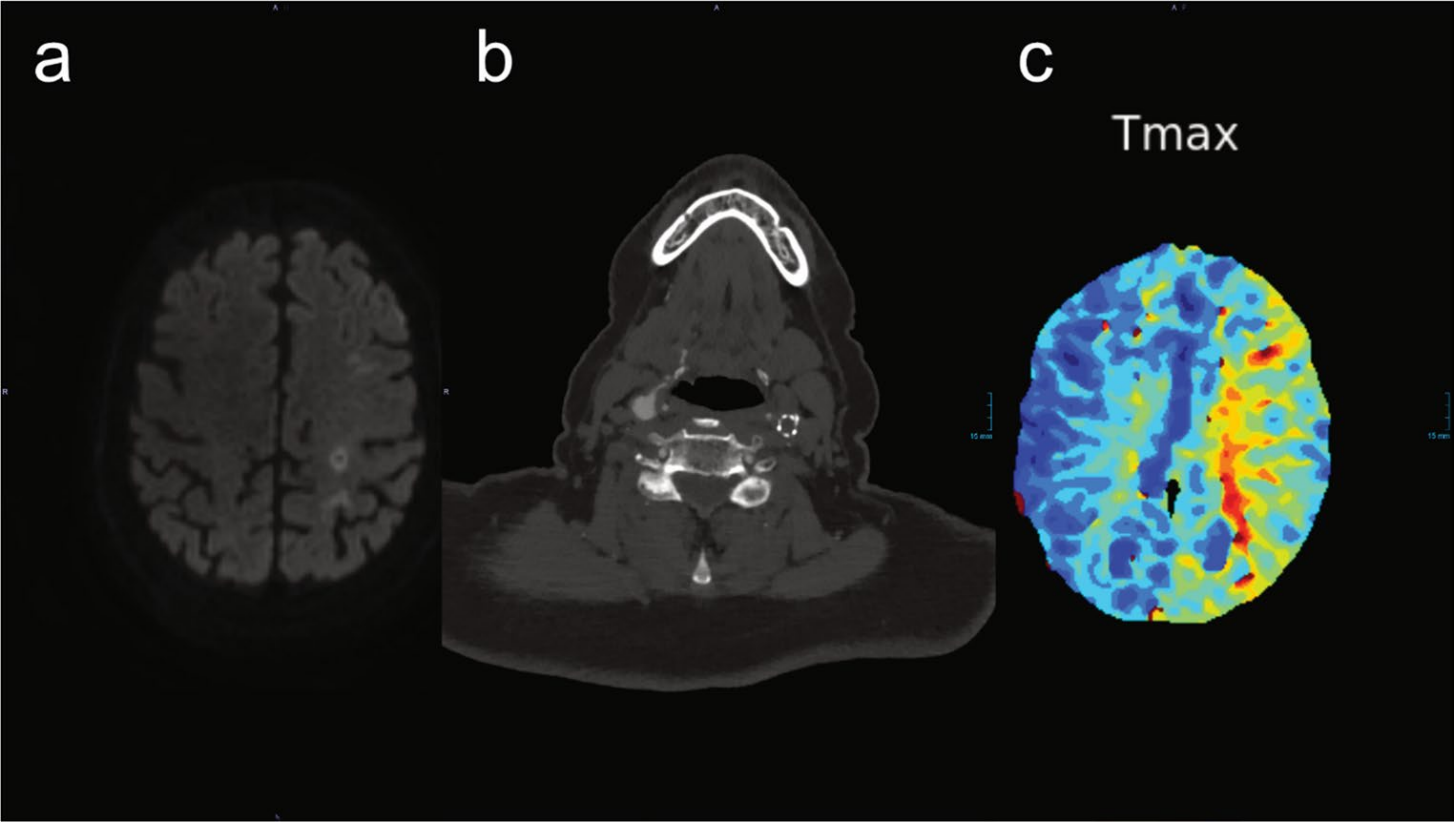
MRI and CTA on admission showing (**a**) acute left watershed infarctions and (**b**) stent occlusion with (**c**) remarkable perfusion impairment in CT-P

**Fig. 3:**
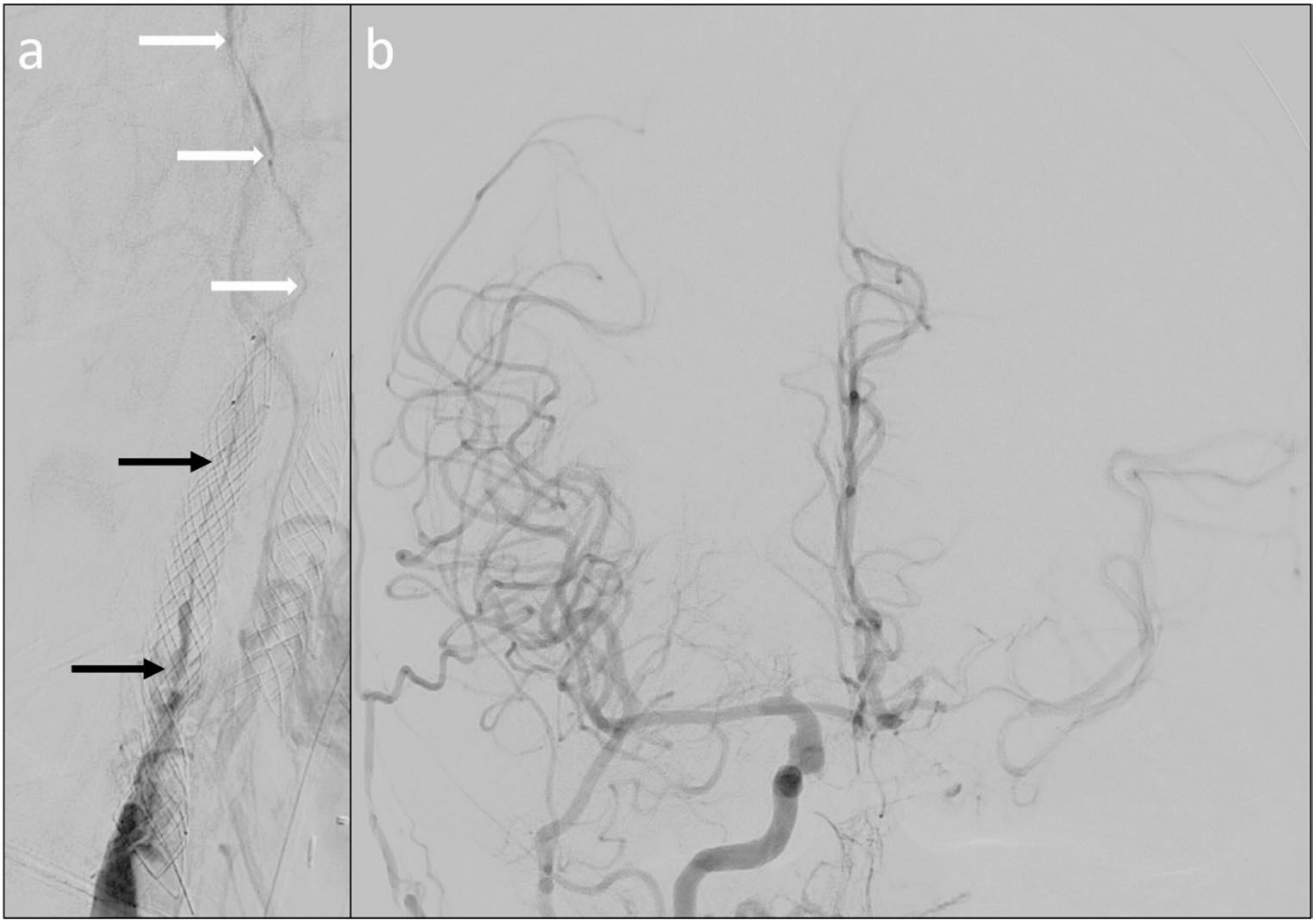
DSA revealing (**a**) near-occlusion within the carotid stent (CASPER, black arrows) and subsequent filiform remaining lumen (white arrows). (**b**) DSA of right ICA showing impaired collateralization via AComA

MRI and CTP examinations revealed acute watershed infarcts in the left hemisphere and notable perfusion impairment (Fig. 2). Digital subtraction angiography (DSA) demonstrated a long-segment chronic occlusion of the left ICA stent (Fig. 3) due to intimal hyperplasia, accompanied by a questionable filiform residual lumen extending to the cavernous segment of the ICA in terms of near-occlusion. Moreover, insufficient collateralization via AComA and PCOM was shown. After thorough evaluation of the potential risks and benefits through multidisciplinary team review, we opted to proceed with an interventional recanalization procedure. The patient received dual antiplatelet therapy comprising aspirin and clopidogrel, and oral anticoagulant therapy was discontinued.

## Treatment

**Fig. 4:**
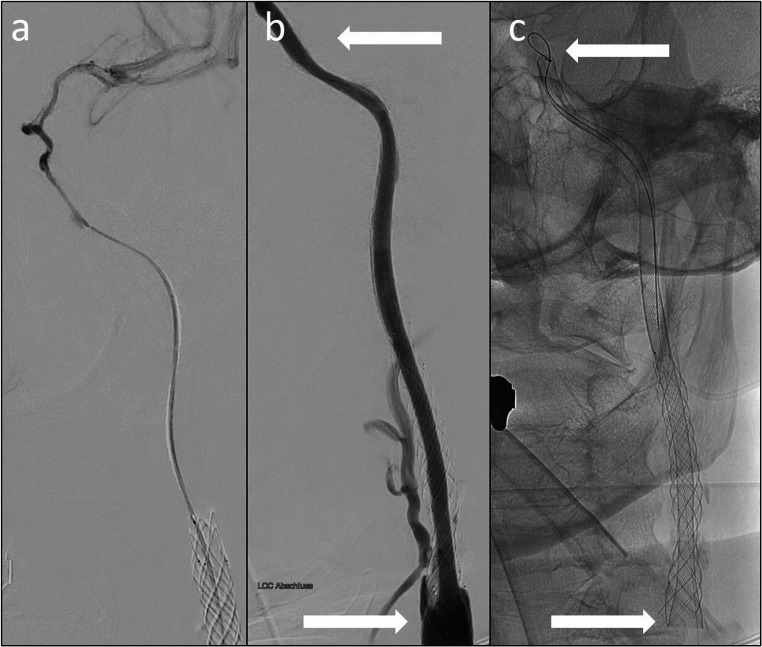
(**a**) Verification of true lumen with microcatheter and (**b** and **c**) implantation of two CARESTO-heal stents in telescope technique after PTA (between white arrows)

The procedure was conducted under general anesthesia using a biplane angiography unit (Philips Azurion Clarity, Philips, Netherlands) under full heparinization. Proximal flow arrest was achieved using an 8 F balloon guide (Walrus, Q’Apel Medical, Fremont, California, USA). The intervention involved careful navigation of the occluded ICA up to the carotid siphon (Fig. 4) using a 0.021 microcatheter (Progreat, Terumo, Japan) and microwire (Synchro, Stryker, CA) to confirm true lumen. After angioplasty with a 2.5 mm balloon from the origin of the ICA up to the cavernous segment (Ryujin, Terumo, Japan), stent angioplasty followed, starting at the proximal end of the former stent, with two self-expandable CARESTO heal stents in a telescopic technique (CARESTO heal 8 × 40 mm proximal and 8/6 × 35 mm distal) under continuous flow arrest and subsequent proximal aspiration. Finally, a PTA of the stents with a 5 mm balloon (Ryujin, Terumo, Japan) resulted in full restoration of the ICA lumen (Fig. 4). The cortical branches were dilated, so strict blood pressure control was aimed at 100 mmHg systolic to avoid hyperperfusion syndrome.

## Outcome and follow-up

**Fig. 5:**
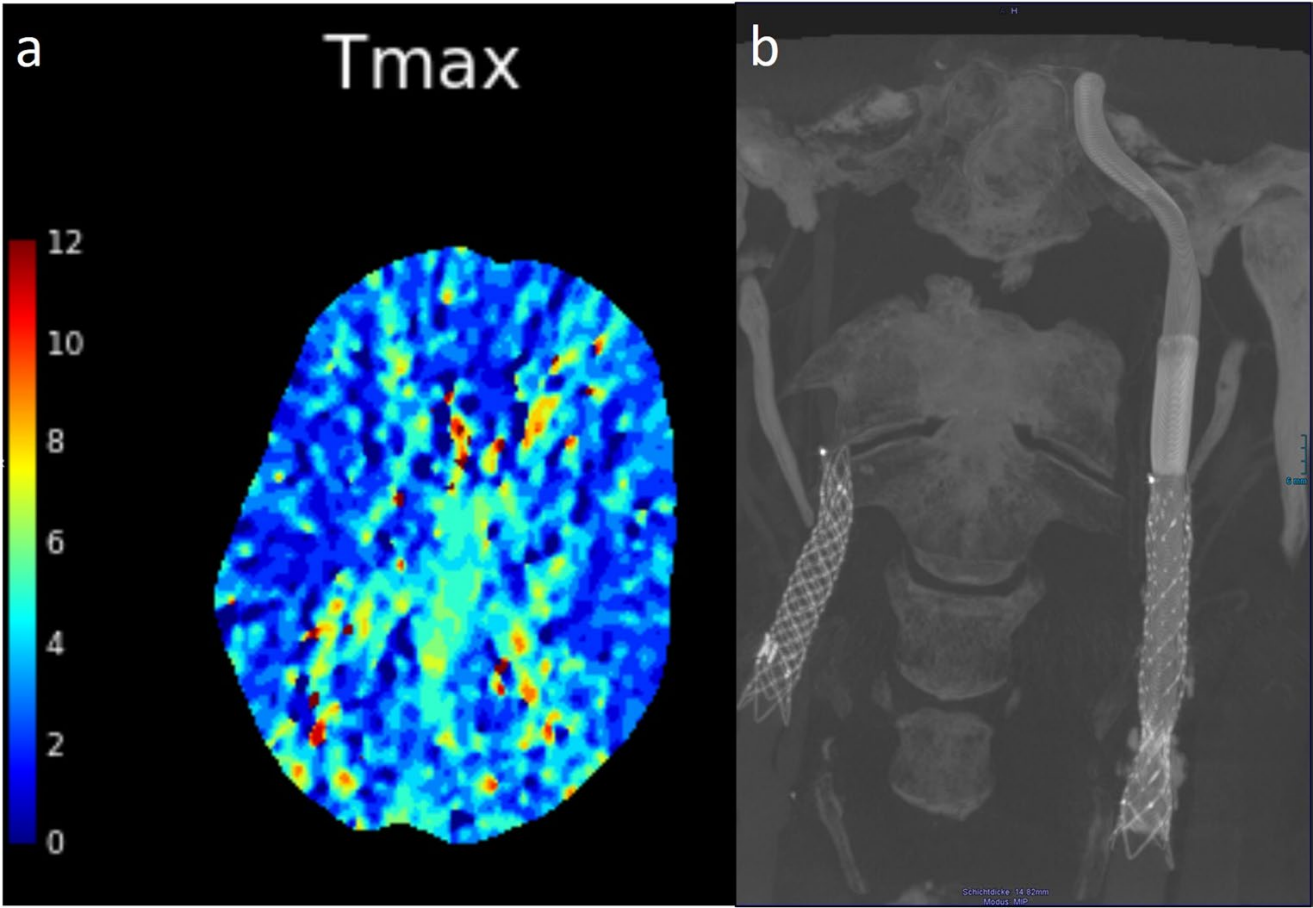
(**a**) CTP 24 h after stenting showing complete normalization of left hemisphere perfusion. (**b**) 6w FU with IV conebeam-CT showing free perfusion of the overlapping CARESTOs

Post-intervention, the patient exhibited no neurological deficits but reported significant headaches. Systolic blood pressure was consistently maintained low for 48 h to prevent hyperperfusion injury. CTP 24 h after stenting showed normalization of left hemisphere perfusion (Fig. 5). At discharge after six days, the patient’s headaches had disappeared, and she had no neurological deficits. She received triple therapy, including dual antiplatelet therapy and reduced oral apixaban for six weeks. Six-week follow-up IV flat-panel CT showed freely perfused CARESTO heal stents without intimal hyperplasia with the patient in good clinical condition.

## DISCUSSION

Carotid artery stenting (CAS) for high-grade stenosis of the ICA is a safe and established procedure [1]. Interventional treatment of chronic ICA occlusions and near-occlusions has been rarely reported [2]. Existing guidelines do not provide clear recommendations for the treatment of chronic ICA occlusion. Nonetheless, there are promising results for selected patients regarding interventional treatment for ICA occlusions or near occlusions, particularly in cases of failure of best medical treatment and hemodynamic relevance [3–5]. However, especially the tortuous course of the petrous and cavernous ICA poses a particular challenge for endovascular vessel reconstruction which could be achieved with the newly designed CARESTO heal.
